# Carbon-Based Composites for Oxygen Evolution Reaction Electrocatalysts: Design, Fabrication, and Application

**DOI:** 10.3390/ma17102265

**Published:** 2024-05-11

**Authors:** Chang Gao, Haiyu Yao, Peijie Wang, Min Zhu, Xue-Rong Shi, Shusheng Xu

**Affiliations:** School of Material Science and Engineering, Shanghai University of Engineering Science, Shanghai 201620, China

**Keywords:** carbon-based materials, oxygen evolution reaction, metal-free, support, core–shell, rational design

## Abstract

The four-electron oxidation process of the oxygen evolution reaction (OER) highly influences the performance of many green energy storage and conversion devices due to its sluggish kinetics. The fabrication of cost-effective OER electrocatalysts via a facile and green method is, hence, highly desirable. This review summarizes and discusses the recent progress in creating carbon-based materials for alkaline OER. The contents mainly focus on the design, fabrication, and application of carbon-based materials for alkaline OER, including metal-free carbon materials, carbon-based supported composites, and carbon-based material core–shell hybrids. The work presents references and suggestions for the rational design of highly efficient carbon-based OER materials.

## 1. Introduction

In order to deal with the issues caused by the fossil fuel energy system, such as energy security and climate change, the development of sustainable clean energy is imperative. Hydrogen is regarded as an ideal secondary energy carrier due to its high energy density. Hydrogen production by electrolysis uses green electricity in the form of solar and wind energy and plays a crucial role in the storage, transformation, and efficient utilization of clean energy [1,2,3]. However, improving the efficiency of hydrogen production and controlling the cost of hydrogen production have been bottlenecks that have restricted the large-scale application of related technologies.

Techniques for hydrogen production through the electrolysis of water include alkaline water electrolysis (AWE [4,5,6]), proton exchange membrane water electrolysis (PEMWE) [7,8], anion exchange membrane water electrolysis (AEMWE) [9], and high-temperature solid oxide water electrolysis (SOWE) [10]. Among them, AWE has been successfully commercialized. However, AWE still faces several problems, such as environmental pollution, equipment corrosion, and low hydrogen production efficiency (59~70%). The oxygen evolution reaction (OER), a half-reaction of hydrogen production by water electrolysis, involves four-electron reaction kinetics. Generally, the OER follows the adsorbate evolution mechanism (AEM). The reaction occurs in alkaline solutions according to Equations (1)–(4):

* + OH^−^ → OH* + e^−^ (1)

OH* + OH^−^ → O* + H_2_O + e^−^ (2)

O* + OH^−^ → OOH* + e^−^ (3)

OOH* + OH^−^ → * + O_2_ + H_2_O + e^−^ (4)

The * means the free surface site. The thermodynamic limiting potential U_L_ is calculated by Equation (5):

U_L_ = max{∆G_1_, ∆G_2_, ∆G_3_, ∆G_4_}/e (5)

However, thermodynamic constraints on the scaling relationship between the Gibbs free energies of *OOH and *OH result in a minimum theoretical overpotential of 370 mV for the optimal catalyst [11]. For oxides-based electrocatalysts, an OER mechanism based on oxygen redox chemistry, namely, the lattice oxygen oxidation mechanism (LOM) [12], has been reported recently. The LOM pathway includes five steps [13]:

*O_l_ H + OH^−^ → *O_l_ + H_2_O + e^−^ (6)

*O_l_ + OH^−^ → *O_l_ OH + e^−^ (7)

*O_l_ OH + OH^−^ → *O_l_ O + H_2_O + e^−^ (8)

*O_l_ O → O_vacancy_* + O_2_ (9)

O_vacancy_* + OH^−^ → *OH + e^−^ (10)

where O_l_ and O_vacancy_* denote the lattice oxygen atoms and lattice oxygen vacancy site, respectively. LOM can bypass O-O bond formation and break the limit of the proportional relationship.

**Figure 1:**
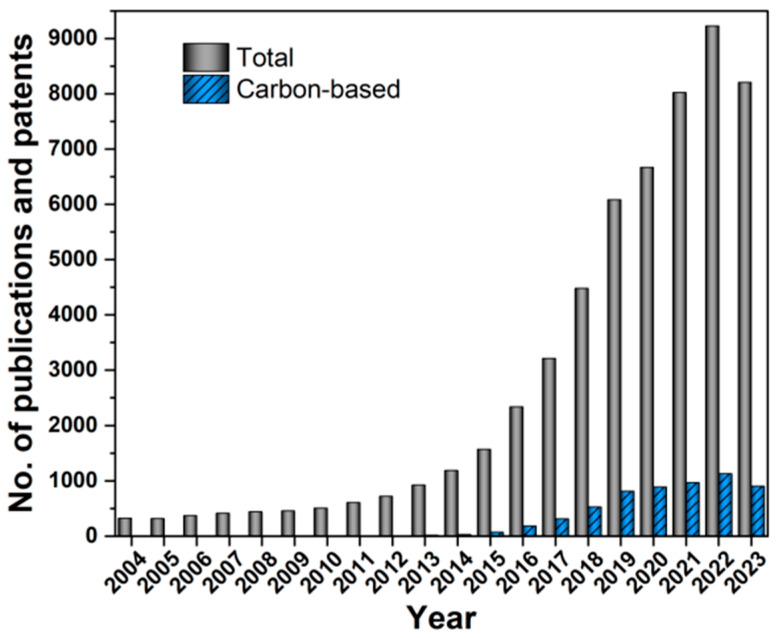
Papers and patents published over the past 20 years that cover oxygen evolution reaction, water splitting, and carbon. Data from the Web of Science.

OER is the bottleneck of hydrogen production by water electrolysis [14]. OER also plays a vital role in the performance of other clean energy conversion and storage devices, such as metal–air batteries and CO_2_ reduction [15,16]. The present OER electrocatalysts are mostly the precious metal oxides IrO_2_ and RuO_2_, limiting their large-scale application. In order to improve hydrogen production efficiency and reduce production costs, active and low-cost OER electrocatalysts have been developed. To date, nonprecious metal oxides, phosphides, sulfides, and alloys have been reported as efficient OER electrocatalysts [17,18]. The OER process of some perovskite electrocatalysts and NiFe-based (oxy)hydroxide follows the LOM mechanism [12]. For RuO_2_, the lattice oxygen is not involved in OER [12]. A comprehensive understanding of the oxygen redox in LOM and possible characterization techniques that can be used to identify the oxygen redox are reviewed in Ref. [19]. After years of effort, significant progress has been made in the design, preparation, and application of high-performance OER materials, including carbon-based materials (Figure 1) [20,21,22,23]. Efficient nonprecious metal oxides, phosphides, sulfides, and alloys can typically reach a current density of 10 mA cm^−2^ at an overpotential <300 mV in 1.0 M KOH [20,21,22,23].

In 2011, Liang et al. [24] fabricated carbon-based materials for effective OER electrocatalysts. They reported a hybrid material consisting of Co_3_O_4_ nanocrystals grown on reduced graphene oxide (rGO) to be an efficient bi-functional catalyst for the oxygen reduction reaction (ORR) and OER. Although Co_3_O_4_ or rGO alone show poor catalytic activity, their hybrid is highly active for OER. The N doping of rGO further boosts OER performance [24]. Inspired by these results, researchers have performed many relevant works and made significant progress [25]. Common electrocatalysts for carbon-based OER materials include metal-free carbon or polymer-based materials, such as covalent organic frameworks (COFs) [26], porous organic polymers (POPs) [27], and composites of carbon and nonprecious metal-containing composite, such as CoP/carbon-cloth (CC), MCoP/CC [28], O-Co-N/C [29], and others [30].

The following review summarizes the research progress and achievable performance levels of carbon-based composites for OER. It aims to provide references and guidance for developing nonprecious metal anode materials for the alkaline electrolysis of water. If the electrocatalyst can work at high current density (HCD), the performance of the electrocatalyst at HCD will be discussed in a separate subsection.

## 2. Design of Carbon-Based Composites

### 2.1. Theoretical Design

With the development of computational methods, especially the in-depth integration of big data and artificial intelligence, various computational methods have been employed to reduce the cost of catalyst development. Computational methods such as density-functional theory (DFT), molecular dynamics, and machine learning (ML) are widely employed for catalyst pre-screening. On this basis, materials are synthesized for verification to realize the rational design of high-efficiency OER electrocatalysts [31,32,33,34,35]. On the other hand, due to the complex four-electron reaction mechanism involved in OER, one of the current research goals is to find simple structural descriptors (such as the electronegativity or the radius of an element) to reveal the structure–activity relationship instead of fully investigating the reaction mechanism to achieve rational design and rapid pre-screening.

**Figure 2:**
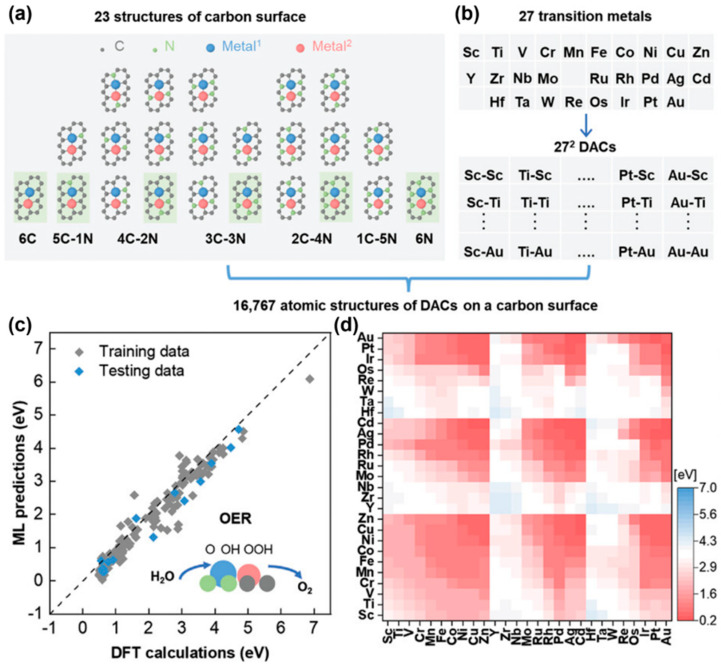
(**a**) A total of 23 types of carbon surface defect structures categorized into seven groups by their N doping level. (**b**) A total of 729 (27 × 27) possible compositional combinations of double-atom electrocatalysts lead to a total of 16,767 (729 by 23) atomic structures on a carbon surface. (**c**) DFT-calculated OER overpotentials versus predictions from the ML model on the training and testing subsets. (**d**) ML-predicted OER overpotentials of all DACs on a symmetric defect structure [36].

Based on the AEM mechanism, Li’s group [36] used a combination of topology-based ML and first-principles calculations to select 511 carbon surface-supported transition metal double-atom catalysts (DACs) out of 16,767 structures with catalytic performance outperforming IrO_2_(110) (Figure 2a,b) for OER. The fitting accuracy of the model for OER activity on the test set was 0.957 (Figure 2c), indicating that the machine learning model can accurately predict the OER catalytic activity of carbon-supported DACs. Figure 2d shows the predicted OER activity results for all DACs. The results showed that carbon surface-supported transition metal double-atom electrocatalysts containing Ni, Cu, Zn, Pd, Ag, Cd, Pt, and Au elements exhibited the best OER catalytic activity [36]. Although DACs containing Au, Zn, and Cd elements can be stably fixed on the defects of a carbon substrate, they have relatively lower stability than other double-atom OER electrocatalysts [36]. Using ML and DFT, Fang et al. [37] screened hundreds of potential dual-metal-site electrocatalysts (DMSCs) and found 10 DMSCs with OER activity superior to RuO_2_. Similarly, Yao et al. [38] performed DFT and ML predictions to systematically investigate the bifunctional OER/ORR activity of 3d transition metal (TM) atom-doped g-C_3_N_3_. ML predictions identified the 3d band center (εd) as the most effective descriptor of the overpotential of OER. Ye et al. [39] employed high-throughput calculations to successfully screen catalytic structures comparable to IrO_2_(110) for OER from a total of 78 edge-anchored single-atom electrocatalysts (E-SACs) based on 26 TM species with three coordination patterns. E-Rh-N_4_-C, in particular, is expected to be an efficiently bifunctional electrocatalyst for OER.

### 2.2. Combined Theoretical and Experimental Design

The joint strategy of computational prediction–experimental verification–computational interpretation is also widely employed. This method can conduct systematic research on a certain catalyst system, achieving mutual verification between calculations and experiments, but it usually requires considerable work. Zhang et al. [40] designed and developed a series of CC self-supported bimetallic phosphides for high-performance OER. Their DFT calculations revealed that Fe/Ni/Mn doping can regulate the electronic properties of CoP, thereby affecting its adsorption ability for key species. On this basis, a series of binder-free MCoP/CC (M = Fe, Ni, or Mn) was synthesized via a hydrothermal–phosphating method. The resulting MCoP/CC achieved high-performance OER in alkaline and simulated seawater solutions with overpotentials at 10 mA cm^−2^ η_10_ lower than 299 mV. A similar combined theory–experiment method has been employed by other groups [41]. For example, Jia et al. [41], Zhang et al. [42], and Li et al. [43] all found that defects played a crucial role in the individual electrocatalytic activity for alkaline OER experimentally and theoretically.

### 2.3. Experimental Design

Various experimental methods have been adopted to improve the activity and stability of OER electrocatalysts. Commonly used strategies include defect engineering, such as heteroatom doping or vacancy introduction [44]; structural control, such as the regulation of morphology and electronic structure [28,45,46,47,48]; and interface engineering [42], such as constructing composites [49,50]. Studies have confirmed that carbon–metal composites can effectively adjust the catalyst’s electronic properties, avoid the metal’s dissolution in the electrolyte, and consequently improve electrocatalysts’ intrinsic activity and stability [51,52,53,54,55]. Regarding the regulation of structural morphology, researchers are committed to the design of hierarchical structures. In particular, nanoarrays, nanospheres, and nanoflowers assembled from one-dimensional nanowires and two-dimensional (2D) nanosheets exhibit excellent OER performance. This is because such hierarchical structures typically have a large specific surface area, exposing abundant reaction sites and sufficient interface sites that provide channels for rapid electron transfer.

## 3. Fabrication and Application

At present, the commonly used preparation method for metal-free COFs and POPs is the solvothermal/hydrothermal strategy [27]. To obtain the conductive carbon support/shell, scientists have widely employed Prussian blue analogs (PBAs) [56,57,58], metal–organic frameworks (MOFs) [59,60], and biomass-derived carbon sources, e.g., chitosan [61], as the carbon matrix precursor due to their adjustable porous structure and a variety of heteroatomic functional groups [62,63,64,65,66,67]. Using heteroatoms with more electrons than C, such as S, N, and P, not only improves conductivity but also induces point defects, promoting OER [68]. In this case, the formation of the (heteroatom-doped) carbon layer is usually through high-temperature calcination. Another simple way of fabricating carbon-based composites is to add carbon-based materials directly during the preparation process, such as CC or carbon nanotubes (CNTs) [69].

In the following sections, we first discuss metal-free carbon-based OER materials. The content of carbon-based composites for OER is organized based on the architecture created by the combined carbon-supported and core–shell architecture.

### 3.1. Metal-Free Carbon-Based Materials

**Figure 3:**
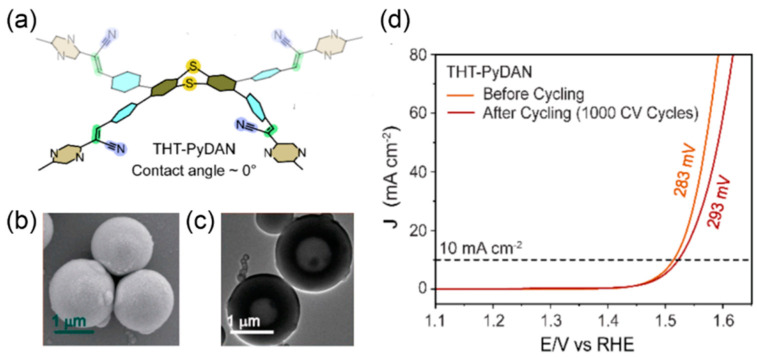
(**a**) Molecular structure, (**b**) SEM image, (**c**) TEM image of THT-PyDAN, and (**d**) its alkaline OER performance before and after 1000 CV cycles [70].

After years of investigation, metal-free COFs and POPs-based materials with comparable activity to IrO_2_/RuO_2_ have been found. Ghosh et al. strategically designed a noble metal-free thiadiazole (TDA) and triazine (Trz) linked porous organic polymer (TDA-Trz-POP) having a N- and S-rich surface [27]. This scrunch paper type TDA-Trz-POP has an η_10_ value of 410 mV and a lower Tafel slope of 104.5 mV deg^−1^ for OER in 1.0 M KOH. Another series of thianthrene-based sp^2^ C = C bonded POPs (Figure 3a) with hollow spherical morphologies (Figure 3b,c), synthesized by Sadhukhan et al. [70], exhibit a superior OER activity. The most efficient bifunctional (hydrogen evolution reaction HER and OER) electrocatalyst THT-PyDAN (Figure 3a) was screened out based on structural tuning to explore the effects of heteroatom incorporation, hydrophilicity, and variations in linker length on electrocatalytic activity. It delivers an η_10_ value of 283 mV in 1.0 M KOH for OER (Table 1, Figure 3d), surpassing all previously reported metal-free bifunctional electrocatalysts. Moreover, THT-PyDAN performs even after 1000 cyclic voltammetry (CV) cycles. A mechanism study by post-catalytic characterization and DFT calculations demonstrated that the intermediates in OER determine the most favorable adsorption site. O * preferentially binds to the S atom of THT-PyDAN, while OH * and OOH * preferentially adsorb on the C atom of the CN group. The potential rate-limiting step is the third step, O* + OH^−^ → OOH* + e^−^ [70]. To overcome the chemical stability issue of metal-free POPs-based structures, a unique strategy is reported [71] by designing robust imidazole-linked POPs via tandem reversible/irreversible bond formation.

In addition to COPs and POPs, other metal-free carbon-based materials with a 2D structure, such as graphitic carbon nitride (g-C_3_N_4_), graphene, and reduced graphene oxide (rGO) are also potential OER electrocatalysts [72]. Among them, biomass-derived carbon materials stand out due to their outstanding features, such as ecological friendliness, abundance, free availability, renewability, high specific surface area, ideal conductivity, controllable porosity, and excellent thermal/chemical stability [73]. Many biomass materials have been used as a carbon source, such as chitin [74], *Platycladus orientalis* tree-cone bio waste [75], and corn stalks [76]. For example, the chitin-derived N-doped carbon material developed by Zhao et al. [74] delivered a comparable alkaline OER performance to iridium and cobalt oxides. Nicotine-containing tobacco [77] and *Euonymus japonicus* leaves [78] were also used as a low-cost raw material to synthesize N-doped ordered mesoporous carbon and micro-layered carbon for OER, respectively. The enzyme-modified matter is carbonized in N_2_ before being pyrolyzed with NH_4_Cl in N_2_, resulting in a 3D N-doped catalyst with a large number of hierarchical pores. The OER activity of optimal N/E-HPC-900 has a lower onset potential, a larger Tafel slope, and better durability than RuO_2_ due to its higher degree of graphitization and level of N doping [79]. Keshab Pandey et al. [80] used coffee waste to fabricate porous carbon for HER and OER. When carbonized at 600 °C for 2 h and activated by KOH at 600 °C, the coffee waste exhibited a low OER overpotential (η_10_ = 230 mV).

**Table 1:** Comparison of metal-free and carbon-based supported materials for OER and water splitting by overpotential (η, mV); current density (j, mA cm^−2^); Tafel slope (TS, mV dec^−1^); and stability (Sta., h).

Materials	Morphology	OER			Water Splitting		Electrolyte	Ref.
		η at j	TS	Sta.	U at j	Stab.		
THT-PyDAN ^a^	POPs, hollow sphere	283@10	81	36@20	1.85@10	12@10	1 M KOH	[70]
TDA-Trz-POP	N,S-rich microporous organic polymer powder	410@10	104.5	3@10	2.61@10	N.A.	1 M KOH	[27]
CCW-KOH-600	Coffee waste-derived porous carbon, activated by KOH at 600 °C	230@10	140	24@10	N.A	N.A.	6 M KOH	[80]
g-C_3_N_4_	Layered composition	355@10	46.8	45@10&50&100	N.A.	N.A.	1 M KOH	[81]
FeNi-C_3_N_4_-P/NF	Interfacial hetero-structure of FeNi phosphide/C_3_N_4_ grown on NF	235@100	40.4	55@100	N.A.	N.A.	1 M KOH	[82]
CoFe_0.05_P/CC	Nanorods grown on CC	264@10	102	45@10	1.59@10	24@10	1 M KOH	[40]
Se-NiS_2_/CC	Se-doped NiS_2_ nanosheets grown on CC	343@50	44.3	30@100	N.A.	N.A.	1 M KOH	[83]
W@Ni(OH)_2_/CC	Ni(OH)_2_ nanosheets and tungsten nanograins form a heterogeneous structure on CC	290@10	67	15@1.52 V	N.A.	N.A.	1 M KOH	[84]
d-NiFeP/CC	Array structure grown on CC	185@10	24.56	100@10	1.48@10	50@10	1 M KOH	[85]
TiN@NiO-NiSe_2_/CC	NiO-NiSe_2_ hetero-structured nanosheets shelling over 1D TiN nanoarrays supported by CC	240@10	29	10@10	1.57@10	50	1 M KOH	[86]
FeCo/Co_2_P/Fe_2_P@NPC	FeCo/Co_2_P/Fe_2_P imbedded in N-doped porous carbon	281@10	85	100@10	1.55@10	24@1.58 V	1 M KOH	[87]
FeCoP_2_@NPPC	Phosphide embedded in a N and P co-doped porous carbon	236@10	83	12@1.5 V	1.6@10	16@10	1 M KOH	[88]
NiV_2_P/FeSe/CC	Ni-Fe-V trimetallic phosphorus–selenium composite grown on CC	168@10 282@200	44.3	160@100	1.53@10	N.A.	1 M KOH	[89]
CuMn_0.5_Co_2_O_4_/CC	Nanoneedles of spinel oxides grown on CC	189@10 327@200	76.2	24@10	N.A.	N.A.	1 M KOH	[90]
Fe-NiOOH/CQDs ^a^	In situ activation of Fe-NiOOH nanoclusters on carbon quantum dots	199@10 450@1000	35	80@10	1.5@10	12@500	1 M KOH	[91]

^a^: The anode and cathode use the same electrode in overall water splitting, with the exception of using Pt/C as the anode. N.A.: Not available.

**Figure 4:**
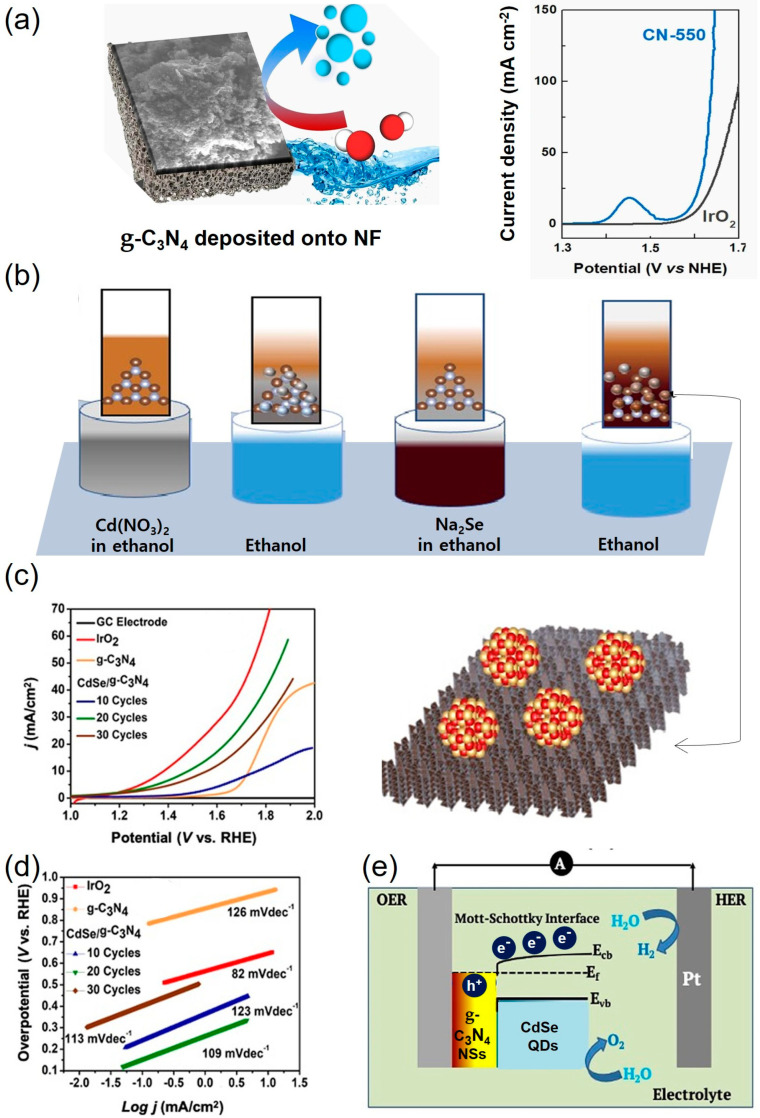
(**a**) The preparation of g-C_3_N_4_ and linear sweep voltammetry (LSV) polarization curves of alkaline OER [81]. (**b**) Schematic illustration of the synthesis of the CdSe QDs/CN heterostructure through the SILAR method [92] and corresponding (**c**) OER LSV, (**d**) Tafel slopes, and (**e**) charge transfer phenomenon at the interface of the heterostructure in electrocatalysis [92].

Graphitic carbon nitride, g-C_3_N_4_, which consists of tertiary amine-linked periodic heptazine units, is also employed as an alkaline OER electrocatalyst. It can be obtained via a facile one-pot method and used for efficient OER without additional post-treatments or co-catalysts. Torres-Pinto et al. [81] assessed the impact of the preparation temperature (450–650 °C) of g-C_3_N_4_ on water-splitting processes for the first time and explained it by different characterization techniques (Figure 4). The unique crystal structure, surface chemistry, and electronic properties of the material prepared at 550 °C led to remarkable OER efficiency with a η_10_ of 355 mV and a Tafel slope of 46.8 mV dec^−1^, defeating the benchmark IrO_2_. It had reduced structural distortion and terminal functional groups, which resulted in improved OER activity and durability. It was stable for up to 45 h, even when the applied current density was increased to 100 mA cm^−2^ for 15 h [81].

Some hybrid materials of biomass-derived carbon and other carbon-based materials, such as CNT and g-C_3_N_4_, also deliver excellent OER performance. For instance, Wu et al. [93] fabricated chitin-derived N-doped carbon (C-Chitin)/g-C_3_N_4_ composites via an ultralow-cost route, including the low-temperature dissolution and subsequent carbonization of chitin biomass. The obtained C-Chitin/g-C_3_N_4_ composites exhibited a well-designed loofa sponge-like three-dimensional (3D) network architecture assembled from many interconnected ultrathin mesoporous nanosheets [93]. Because of the well-designed morphology, a unique mesoporous structure with a high surface density of catalytic active sites, and the combination of N-doped carbon with g-C_3_N_4_, the C-chitin/g-C_3_N_4_ composites had high durability in OER [85]. However, metal-free carbon-based materials are not able to reach an industrial current density at a low overpotential.

### 3.2. Carbon-Based Supported Composites for OER

#### 3.2.1. At Low Current Density (LCD)

Interestingly, in addition to being used as an electrocatalyst directly, g-C_3_N_4_ can also be used as a substrate of supported OER electrocatalysts, such as oxides, phosphides, and sulfide-based materials. The nitrogen atoms in g-C_3_N_4_ have a lone pair of electrons and can change the electron configuration of the supported materials by interfacial charge redistribution. Studies on the Co_3_O_4_/g-C_3_N_4_ nanocomposite [94] and g-C_3_N_4_/Co_3_O_4_/α-Fe_2_O_3_ [95] have proved that a strong coupling interaction is formed at the interface between the g-C_3_N_4_ and the oxide, which acts as an effective electron transport channel and exposes more catalytically active sites, leading to outstanding OER performances with η_20_ values of 170 and 359 mV and Tafel values of 188 and 116 mV dec^−1^, respectively. Following the synthesis of g-C_3_N_4_ NSs, CdSe quantum dots (QDs)/CN heterostructures are created through the successive ionic layer adsorption and reaction (SILAR) process (Figure 4b) [92]. As observed from LSV curves in a 0.1 M KOH electrolyte (Figure 4c), CdSe QDs/CN with 20 SILAR cycles presents outstanding OER activity with η_10_ of 200 mV, which is close to commercially available IrO_2_ and surpasses g-C_3_N_4_ and CdSe QDs. Its Tafel slope of 109 mV dec^−1^ indicates more favorable OER kinetics (Figure 4d). Figure 4e illustrates the charge transfer at the interface of the heterostructure for overall water splitting (OWS) to develop H_2_ and O_2_ [92]. Another work [96] proposed a hierarchical carbon matrix decorated with nitrogen atoms (NC) as a support for obtaining high-performance ORR/OER electrocatalysts based on iron and nickel (Fe/Ni@N–C).

**Figure 5:**
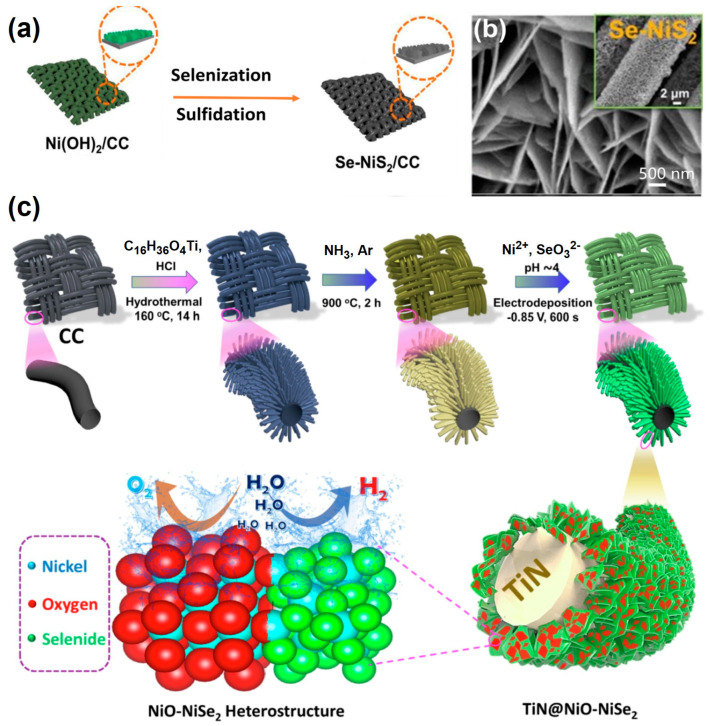
(**a**) Schematic illustration of fabricating Se-NiS_2_ on CC and (**b**) SEM image of Se-NiS_2_/CC [83]. All rights reserved. (**c**) Schematic design for the fabrication of TiN@NiO-NiSe_2_ on CC [86].

Among various carbon-based materials, CC is more widely used than g-C_3_N_4_ and NC for OER because of its excellent conductivity and high cost-effectiveness. The large specific surface area of CC enhances the contact between the electrode material and the electrolyte, and the constructed composites favor the thorough exposure of catalytic sites and charge/mass transfer, thus boosting electrochemical performance. Like a nickel foam (NF) substrate, active materials can be grown on it in situ to decrease the interface impedance. Both Chen et al. [83] (Figure 5a,b) and Kang et al. [84] used a grown-in situ Ni(OH)_2_ nanosheet array on CC as a template. Subsequently, Kang et al. [84] employed magnetron sputtering technology to uniformly deposit tungsten nanoparticles onto Ni(OH)_2_ nanosheets, successfully fabricating a heterostructure of W@Ni(OH)_2_/CC. Chen et al. [83] fabricated three control samples for OER via mild sulfidation or selenidation: NiS_2_, NiSe_2_, and Se-doped NiS_2_ (Se-NiS_2_). Islam et al. [86] successfully developed a thin shell of NiO-NiSe_2_ nanosheets on a template of in situ-grown TiN nanoarrays on CC (TiN@NiO-NiSe_2_/CC) (Figure 5c) for high-performance OER.

By constructing a heterogeneous interface and adopting a 2D nanosheet morphology for the catalyst, the charge transfer, OER kinetics, and ion/gas transport processes are effectively accelerated. The large surface area with abundant heterogeneous interfaces synergistically enhances the OER activity. The conductive substrate facilitates electron transfer, while the in situ integration characteristics favor stable adhesion between the substrate and hybrid catalyst. Additionally, the electronic coupling effect of the heterostructure enhances both the OER kinetics and charge transfer performance and also drives the transformation of the catalyst surface into the active NiOOH layer. First-principal calculations reveal that the transformation to active NiOOH is strongly related to the bond strength [83]. For example, the Ni-S bonds in NiS_2_ are too strong to generate NiOOH, and the Ni-Se bonds in NiSe_2_ are too weak, causing Se to dissolve and leading to structural instability [83]. Meanwhile, the Ni-S bonds and Ni-Se bonds in Se-NiS_2_ are modulated and moderate, resulting in the Se-NiS_2_ surface’s rapid conversion into NiOOH [83]. Consequently, Se-NiS_2_ delivers improved OER activity and stability under alkaline conditions, with η_50_ of 343 mV [83]. During the surface’s evolution into highly active NiOOH, anion leaching could occur. Anion leaching and adsorption onto the reconstructed surface is crucial for enhancing catalytic performance, as the disordered, porous, high surface area skeleton catalyst possesses numerous active sites that could be penetrated by the electrolyte and exhibit bulk activity. In addition, the defects could also promote the surface’s transformation into highly active MOOH. For instance, Chen et al. [85] achieved a binder-free defect-rich NiFeP electrocatalyst (d-NiFeP/CC) by selectively etching Zn atoms from the Zn-NiFeP precursor. Acid etching primarily affected Zn atoms rather than Ni and Fe atoms, introducing surface defects. The introduction of defects not only facilitates the surface’s evolution into highly active NiOOH but also improves its stability. The d-NiFeP/CC exhibits a η_10_ value of only 185 mV with a Tafel slope of 24.56 mV dec^−1^ and stability of up to 100 h in alkaline OER. For OWS, d-NiFeP/CC displays a small cell voltage of 1.486 V to drive a current density of 10 mA cm^−2^.

**Figure 6:**
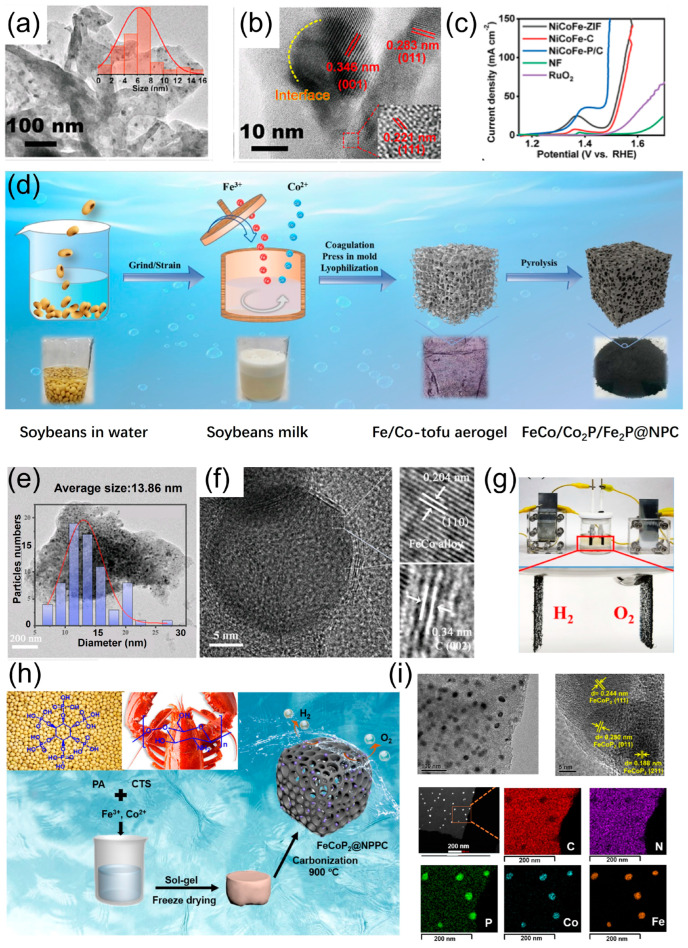
(**a**) TEM (inset: the particle size distribution), (**b**) HRTEM images, and (**c**) alkaline OER performances of NiCoFe-P/C [98]. (**d**) Schematic illustration of the preparation procedure of FeCo/Co_2_P/Fe_2_P@NPC. (**e**) TEM image of FeCo/Co_2_P/Fe_2_P@NPC (inset: the size distribution of nanoparticles), and (**f**) HRTEM images of one nanoparticle. (**g**) Digital photo showing the operating state of the as-constructed self-powered overall water-splitting system [87]. (**h**) Synthesis route and (**i**) TEM and HRTEM images of FeCoP_2_/NPPC [88].

In addition to sulfides/selenides, non-noble transition metal phosphides (TMPs) show superior or comparable OER activity to IrO_2_/RuO_2_ due to their unique and tunable electronic properties. The disadvantage of TMPs in accessible surface area can be compensated for by introducing conductive and porous carbon materials to improve mass/charge transfer efficiency. Numerous attempts have been made to develop effective TMP/carbon composites for OER. Amongst various TMP phases, multi-metal centered TMPs such as alloyed FeCo phosphides exhibit enhanced OER activity [97]. Liu et al. [98] fabricated a composite of NiCoFe-P/C with 0-dimensional (0D) NiCoFe-P QSs supported by 2D N-doped C (Figure 6a,b) via the carbonization and phosphorization of NiCoFe-ZIF precursors. The prepared NiCoFe-P/C 0D/2D heterostructure had excellent alkaline OER activity with η_100_ of 257 mV (Figure 6c). Safer substitutes, such as phytic acid (PA), are being explored to replace toxic phosphorus sources, such as NaH_2_PO_2_, P(SiMe_3_)_3_, tri-noctylphosphine, and elemental phosphorus. Wang et al. [87] initiated a one-pot strategy for the green, facile, and mass production of a highly active and stable catalyst for OER/ORR/HER through the preparation of Fe/Co cross-linked tofu gel and its subsequent pyrolysis (Figure 6d). Despite the free use of additional N/P precursors or pore-forming agents, the as-prepared materials comprised highly dispersive FeCo-rich phosphide nanoparticles and a porous N,P co-doped carbon framework inherited from the tofu skeleton (Figure 6e,f). The as-fabricated electrocatalysts exhibited remarkably enhanced trifunctional activities, with η_10_ values of 281 mV vs. 341 mV (RuO_2_), 353 mV (Fe_2_P@NPC), 376 mV (Co_2_P@NPC), and 453 mV (NPC), along with better long-term stability than the benchmark RuO_2_ catalyst. Furthermore, the as-constructed alkaline electrolyzer required only a small voltage of 1.55 V@10 mA cm^−2^, which outperforms nearly every biomass-derived electrocatalyst ever reported, and that of noble metal catalyst-based electrolyzers (1.72 V@10 mA cm^−2^ for Pt/C-RuO_2_) (Figure 6g) [87]. A similar green synthesis strategy was employed by Wang et al. [88] (Figure 6h,i) and Zhang et al. [91], who used biomass-derived N-containing chitosan as the carbon source and PA as the precursor of both phosphorus and carbon. After being stirred to form a chelated structure, freeze-dried, and carbonated, the obtained dual-TMP nanoparticles embedded in (N, P) co-doped porous carbon exhibited excellent alkaline OER performances. FeCoP_2_@NPPC [88] and Ni_0.8_Fe_0.2_P-C [99] require overpotentials of 236 and 242 mV for OER reaching 10 mA cm^−2^ in a 1.0 M KOH solution, respectively. The latter can work stably for over 100 h [99].

Carbon paper (CP) [100,101,102], rGO [103], and graphene [104,105,106] are also used as the support. The heterostructure directly grows on CP with a high surface area and excellent conductivity, ensuring outstanding electrode performance. For example, a Ni_0.70_Fe_0.10_V_0.20_S_2_ core encapsulated within an amorphous NiFe hydroxide shell supported on CP presents excellent OER performance in a 1.0 M KOH electrolyte, with η_10_ of 204 mV and a Tafel slope of 39 mV dec^−1^ [100]. These superior characteristics are primarily attributed to the synergistic effects between the core–shell structure, the high electronic conductivity of the core, the presence of additional active sites on the amorphous shell surface, and the strong adhesion between the active materials and conductive support. The incorporation of metallic V expedites electron transfer during the OER process, while Fe doping introduces 3d states near the Fermi level, thereby enhancing electronic conductivity. The in situ-generated amorphous iron hydroxide possesses many high-valent active sites, favoring the adsorption of negatively charged reaction-involved species like OH^−^ and HO_2_^−^.

#### 3.2.2. At HCD

Although much progress has been made in developing LCD OER electrocatalysts, most electrocatalysts perform well only at relatively low current densities, which do not meet the requirements of industrial electrochemical applications. For practical production applications, stable operation at high current densities (≥500 mA cm^−2^) is imperative [12]. At HCD, the accumulation of a large number of bubbles will greatly slow down the charge and mass transfer of the electrode, reduce the exposure of the active site, and lead to high overpotential, which sets stricter demands on HCD OER electrocatalysts. The high surface area and high porosity of carbon-based materials favors the diffusion of oxygen molecules [82].

Studies have shown that grown-in situ active OER materials on CC with honeycomb-like morphologies composed of nanosheets [89], nanoneedle arrays [75], and nanofollowers assembled by nanosheets/nanowires [107,108] have excellent HCD OER performance. As in the LCD scenario, CC self-supported multi-metal center composites usually perform better than single-metal systems [109]. That may be because the differences in the enthalpy values of the metal centers induce the formation of the heterojunction, causing the rearrangement of interface electrons and resulting in electron-enriched and electron-deficient regions. This facilitates the adsorption of H^+^ and OH^−^/H_2_O, thereby promoting HER and OER activity [110]. For instance, Qin et al. [89] used in situ-grown V-MOF on CC as the precursor, followed by the introduction of Ni and Fe components through electrodeposition, synchronized phosphorization, and selenization treatments, ultimately yielding a Ni-Fe-V ternary metal phosphoselenide composite (NiV_2_P/FeSe@CC). The obtained hierarchical porous 3D CC-supported honeycomb-like composite delivered η_10_ and η_200_ of only 168 and ~282 mV in an alkaline environment, respectively. The assembled electrolyzer required a voltage of 1.53 V at 10 mA cm^−2^ and exhibited outstanding stability for 160 h at 100 mA cm^−2^. Also, the in situ-grown mesoporous CuMn_0.5_Co_2_O_4_ nanoneedle array on CC (CuMn_0.5_Co_2_O_4_/CC) achieved η_10_ and η_200_ of 189 and ~327 mV in 1 M KOH, respectively, outperforming Co_3_O_4_/CC, CuCo_2_O_4_/CC, and some other single-metal oxide systems [90]. Similarly, the CC self-supported follower-like hierarchical dual-metal sulfides, e.g., an FeS/Co_3_S_4_/CC heterostructure [107] or a non-metal-doped sulfide, e.g., V,P-CoS_2_/CC [108], also showed improved performance compared with the single component. 

**Figure 7:**
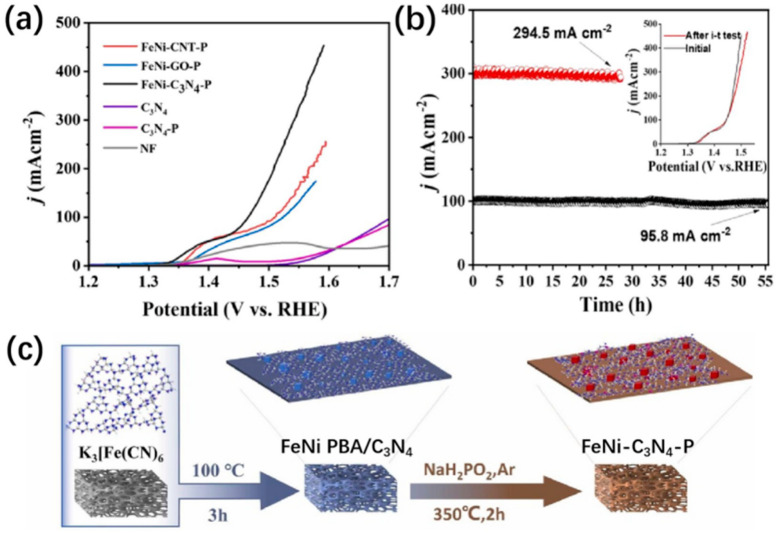
(**a**) OER LSV curves. (**b**) i-t chronoamperometric tests for FeNi-C_3_N_4_-P at overpotentials of 245 mV and 320 mV for 28 h and 55 h in turn. Inset is the comparison of polarization curves of FeNi-C_3_N_4_-P after the i-t test. (**c**) Schematic illustration for the formation of NF-supported FeNi-C_3_N_4_-P [82].

Zhang et al. [82] compared the effect of different carbon substrates on catalytic performance (Figure 7a). They found that C_3_N_4_ was a better substrate than CNT and GO, possibly due to the presence of N. In addition, C_3_N_4_ and P-doped C_3_N_4_ (C_3_N_4_-P) loaded on NF displayed little catalytic activity below 1.55 V. Although Ni phosphide may also form in P-doped C_3_N_4_, in which Ni is from the NF support, the amount of Ni phosphide produced is insufficient to catalyze the OER. From the experiments described above, it can be concluded that using K_3_Fe(CN)_6_, C_3_N_4_, NaH_2_PO_2_, and NF is critical for the superior performance of the FeNi-C_3_N_4_-P catalyst. To synthesize the FeNi-C_3_N_4_-P catalyst, FeNi PBA was first produced on a NF substrate in the presence of C_3_N_4_. The obtained material was then subjected to a high-temperature phosphating process (Figure 7c). As expected, the resulting FeNi-C_3_N_4_-P catalyst exhibits excellent OER activity. It shows a small Tafel slope of 40.4 mV dec^−1^, a low overpotential of 235 mV at 100 mA cm^−2^, and remarkable stability (Figure 7b) [82].

In addition to the common *n*D/3D hierarchical structure [111], the *n*D/0D hierarchical structure has also been developed for HCD OER. Carbon quantum dots (CQDs), characterized by their small nanoscale dimensions, effectively prevent corrosion and extend stability [112]. Additionally, CQDs possess favorable hydrophilic and aerophobic properties, aiding in regulating the electrolyte/catalyst interface and facilitating the rapid ingress of reactants and smooth detachment of oxygen bubbles to boost HCD OER further. When used in constructing the composites, CQDs pass on excellent hydrophilic and aerophobic properties to the connected active materials. Recently, Pan et al. [91] reported an in situ activation of Fe-NiOOH nanoclusters on CQDs (Fe-NiOOH/CQDs) to achieve HCD OER. After directly dropping a NiFe-CQD ink solution on the CP, the prepared working electrode demonstrated an in situ self-activation process in OER. It took 50 cycles of CV activation to fulfill the almost complete evolution of MO to a M-OOH active species so that the system reached optimal activity (Tafel slope: 35 mV dec^−1^, η_10_ = 199 mV, η_1000_ = 450 mV with a turnover frequency of up to 5.4 s^−1^).

### 3.3. Carbon-Based Core–Shell Composite

Although carbon substrates can effectively improve conductivity, direct exposure of active materials such as metal compounds to electrolytes can lead to the inevitable corrosion dissolution of metals and structural collapse, resulting in poor stability. A core–shell structure is widely used to improve catalyst stability [113,114,115,116]. Fabricating a carbon shell has the following advantages: (i) conductivity can be greatly improved; (ii) the electronic interaction between the carbon shell and the encapsulated metal can promote the activation of catalytic sites and improve intrinsic activity; (iii) carbon coating can protect encapsulated metals from corrosion caused by direct contact with electrolytes, effectively enhancing the stability of the structure. Based on this, extensive research has been conducted on carbon-coated structures for high-performance OER [117]. Note that a suitable thickness of carbon shell is required for optimal OER performance.

#### 3.3.1. At LCD

As previously mentioned, TMPs deliver superior OER activity. Great efforts have been made to develop not only TMP/carbon but also TMPs@carbon [118,119] electrocatalysts, creating a viable alternative to IrO_2_/RuO_2_-based electrocatalysts. Structural regulation is essential for realizing efficient and stable OER catalysis. Lv et al. [120] reported an effective strategy to enhance the performance of core–shell Co_2_P@NC electrocatalysts via secondary metal (e.g., Fe, Ni, Mo, Al, Mn) doping (referred to as M-Co_2_P@M-N-C). Under a hydrogen atmosphere, the transition metal-doped organic–inorganic phosphonate cobalt (tm-melamine/TM-HEDP-Co) precursor introduces secondary metal into the Co_2_P core during high-temperature pyrolysis, simultaneously forming a M-N-C shell with highly active M-N_4_ sites. The addition of Fe enhances the activity of the Co_2_P core in HER/OER by optimizing the electronic structure, while well-constructed Fe-N_4_ active sites in the Fe-N-C shell effectively promote OER/ORR. Thereby, the Fe-Co_2_P@Fe-N-C electrocatalyst exhibits the best catalytic activity among the M-Co_2_P@M-N-C electrocatalysts, achieving a current density of 10 mA cm^−2^ at 1.58 V for OWS (Table 2). This outstanding performance is closely associated with the subtle synergistic effect between the highly active components (the Fe-doped Co_2_P core and Fe-N-C shell) and the durable carbon shell. The stable Fe-N-C shell plays a vital role in rapid electron transport and effectively prevents the corrosion of the Fe-Co_2_P core in alkaline electrolytes.

Carbon-encapsulated alloys, oxides, carbides, and sulfides (alloys@carbon [60], oxides@carbon [97], carbides@carbon [121]), sulphides@carbon [122]) are other representative core–shell electrocatalysts for OER. Alhakemy et al. [123] employed a facile one-step approach to fabricate a composite with CoFe_2_O_4_ (CFO) as the core and carbon spheres (CSs) as the shell. The incorporation of CFO introduced additional catalytic active sites for OER, endowing the CFO/CS with low overpotential and charge transfer resistance during the OER process. After extended stability tests, the CFO@CSs hybrid retained its spherical structure, with CFO nanoparticles uniformly dispersed within the CSs, demonstrating excellent structural stability. Abbas et al. [121] encapsulated iron carbide (Fe_3_C) nanoparticles within a N-doped graphite carbon shell through a straightforward carburizing ion process. The graphite carbon shell significantly enhanced the activity and stability of the Fe_3_C@C and Fe_3_C@C-N electrocatalysts compared to the bare Fe_3_C nanoparticles. Fe_3_C@C-N exhibited markedly improved activity and stability in OER, attributable to the synergistic effect between the active core and the stabilizing shell structure. Note that the Fe-N sites in Fe_3_C@C-N are not OER-active centers. However, N doping increased the quantity of Fe_3_C in the catalyst and hence surpassed the overall performance of Fe_3_C@C.

**Table 2:** Comparison of core–shell carbon-based materials for OER and water splitting by overpotential (η, mV); current density (j, mA cm^−2^); Tafel slope (TS, mV dec^−1^); and stability (Sta., h).

Materials	Morphology	OER			Water Splitting ^a^		Electrolyte	Ref.
		η at j	TS	Sta.	U at j	Stab.		
Fe-Co_2_P@Fe-N-C	Core–shell nanostructure; Fe-doped Co_2_P core and Fe-N-C shell	300@10	79	24@50	1.58@10	10	1 M KOH	[120]
CFO@CSs	Carbon-coated spherical structure; core: CoFe_2_O_4_	390@10	57.75	6@10	1.87@10	20@10	1 M KOH	[123]
Ni@PMMA-BM	Carbon-coated honeycomb-like structure	263@10	34	28@10	N.A.	N.A.	1 M KOH	[124]
Fe_3_C@C–N	Core–shell nanostructure; core: Fe_3_C; shell: nitrogen-doped graphene-like layers	469@10	89	13.8@1.7 V	N.A.	N.A.	0.1 M KOH	[121]
Fe/Fe_3_C@C/CNT	Carbon-coated Fe/Fe_3_C nanoparticles anchored to CNTs	292@10	29	12@50	N.A.	N.A.	1 M KOH	[125]
Ni_0.70_Fe_0.10_V_0.20_S_2_-AH/CP	Core–shell trimetallic heterostructures grown on carbon paper	204@10	39	24@10	N.A.	N.A.	1 M KOH	[100]
CoP/DCS	CoP nanoparticles encapsulated in a rich-defect carbon shell	251@10	81.6	72	1.49@10	24@10	1 M KOH	[117]
S/N-CMF@Fe_x_Co_y_Ni_1−x−y_-MOF	Core–shell nanostructure; core: S/N-doped carbon mesoporous fibers; shell: ternary metal centers MOFs	296@10 377@200	53.5	100@10	N.A.	N.A.	1 M KOH	[68]
(V_2_O_3_−CoFe_2_O_4_)@C/NF ^a^	V_2_O_3_-decorated spinel CoFe_2_O_4_ with carbon-encapsulated nanosheets grown on NF	226@10 310@500	56	80@500	1.53@10 1.89@150	80@100	1 M KOH	[18]

^a^: The anode and cathode use the same electrode in WOS with the exception of using Pt/C as the anode. N.A.: Not available.

Besides the composition (C, C-N, C-P, and other combinations), the thickness of the carbon coating is also a key factor in regulating the electron penetration effect and catalytic performance for interfacial heterogeneous catalytic reactions. Although carbon coating can improve catalytic stability, an extremely thick carbon layer can weaken the electron penetration effect and hinder the improvement of intrinsic activity. DFT calculations reveal that as the graphene layer of the carbon shell increases, the electron transfer from the metal core to the outermost carbon shell rapidly decreases [124]. Therefore, it is necessary to precisely control the thickness of the carbon coating through a convenient and effective preparation method, which is the key to optimizing the electron penetration effect and achieving a balance between activity and stability.

**Figure 8:**
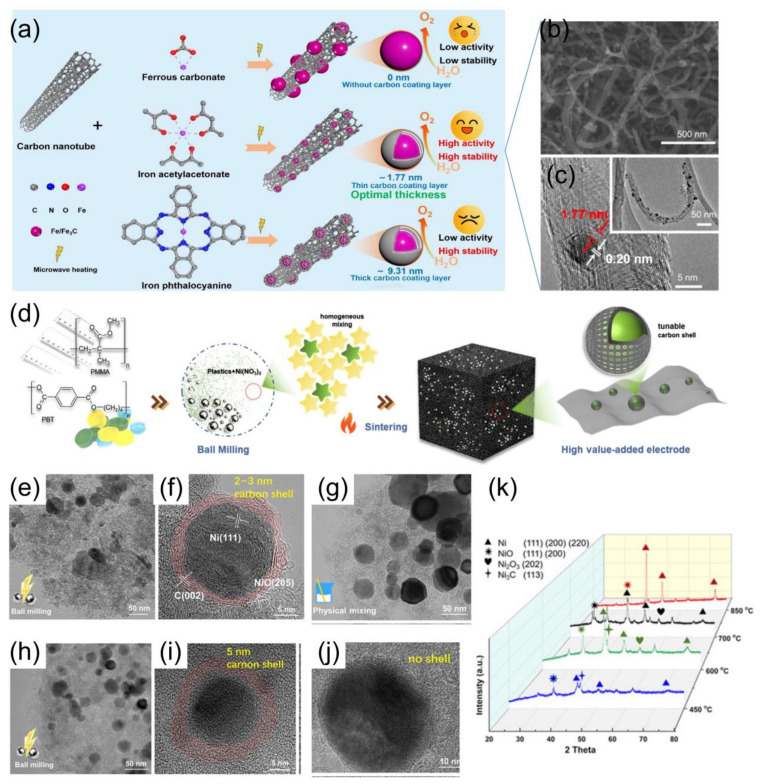
(**a**) Schematic illustration of fabricating Fe/Fe_3_C-C@CNT, Fe/Fe_3_C-A@CNT, and Fe/Fe_3_C-P@CNT [125]. (**b**) SEM and (**c**) TEM of Fe/Fe_3_C-A@CNT [125]. (**d**) Schematic illustration for the mechanochemical synthesis of the metal/metal oxides@carbon [124]. TEM images of (**e**,**f**) Ni@PMMA-BM (1 h), (**h**,**i**) Ni@PMMA-BM-3 h, and (**g**,**j**) Ni@PMMA-PM. “3 h” represents the ball milling treatment [124]. (**k**) XRD of Ni@PMMA-BM at 450 °C–850 °C [124].

The differences in carbon content among different carbon sources significantly impact the growth process of heterostructures. Metal–organic compounds with high carbon content can promote the formation of carbon coatings, while inorganic metal compounds with lower carbon content tend to create thin or no carbon coatings. Using iron acetylacetonate (Fe(C_5_H_7_O_2_)_3_), ferrous carbonate (FeCO_3_), and iron phthalocyanine (C_32_H_16_FeN_8_) as both iron and carbon precursors, Gao et al. [125] found that in situ fast carbonization of acetylacetonate iron based on CNT-induced rapid microwave heating can obtain Fe/Fe_3_C-A@CNT with a suitable carbon layer thickness and hence effectively prevent the aggregation of iron-based compounds and promote the uniform dispersion of Fe/C heterostructures (Figure 8a). A higher carbon content in metal–organic compounds makes it easier to form a carbon coating on the metal surface during carbonization, as observed with acetylacetonate iron and phthalocyanine iron, where the thickness is directly proportional to the carbon content in the metal source [125]. Experimental results demonstrate that an Fe/Fe_3_C-A@CNT sample with a surface carbon layer of ~1.77 nm thickness exhibits a lower overpotential (η_10_ = 292 mV) and a smaller Tafel slope (29 mV dec^−1^) than samples with thicker carbon layers (>9 nm) (Fe/Fe_3_C-P@CNT and FeACAO@CT) and samples without a carbon layer (Fe/Fe_3_C-C@CNT) [125]. In another work, Zhao et al. [124] found that the length of ball milling treatment (Figure 8d–f,h,i) also affected the thickness of the carbon shell when they employed a mechanochemical approach with plastic waste as the precursors to synthesize Ni@C electrocatalysts with tunable core–shell structures, octahedral-like crystal (OLC) morphology, and abundant surface defects. The ball milling process effectively facilitates the uniform dispersion of the metal precursor within the plastic matrix, resulting in an adjustable active core–shell structure. The metal species, encapsulated by carbon shells derived from plastic, are uniformly embedded in a highly porous carbon support. OER electrocatalysts based on transition metals (such as Co, Ni, Mn, and Fe) @C exhibited significantly enhanced OER kinetics than commercial IrO_2_, with performance following the order Ni > Co ≈ Fe > Mn. A carbon shell with a controllable thickness alters the permeability of reactants/electrolytes by eliminating specific adsorption sites on the metal surface. The optimal Ni@PMMA-BM prepared by the mechanochemical method achieved a current density of 10 mA cm^−2^ at 263 mV in 1.0 M NaOH. XRD patterns of the Ni@PMMA-BM samples sintered at different temperatures (Figure 8k) demonstrate that as the temperature increased, Ni_3_C and Ni oxides gradually disappeared and finally all converted to metallic Ni. That is, ball milling promotes the physical mixing and full contact of the carbon precursor with nickel nitrate and also the in situ formation of a carbon layer outside the Ni particles during the annealing process under N_2_ at 850 °C (Figure 8k).

#### 3.3.2. At HCD

Similar to OER at LCD [65], a suitable carbon shell thickness is required for optimal OER performance at HCD. An increase in carbon layer thickness and crystallinity could attenuate the activity of potential active metal sites and hinder electron transport, as confirmed by Wang et al. [126]. They found that the synthesized high-entropy alloy (HEA) CoNiCuMnAl@C nanoparticles derived from multi-metallic MOFs [126] at a calcination temperature of 900 °C had a denser carbon layer and higher crystallinity and showed worse catalytic performance than those with a thinner carbon layer. The optimal CoNiCuMnAl@C/NF (deposited film on NF via electrophoretic deposition) with a novel core–shell nanostructure (core: a face-centered cubic HEA, shell: an extremely thin carbon layer) substantially promoted OER in 1.0 M KOH, exhibiting η_200_ of ~279 mV. Immersion in a 1.0 M KOH solution for up to 30 h at 200 mA cm^−2^ demonstrated its enduring OER stability. For comparison, by depositing CoNiCuMnAl@C on commercial carbon paper, the electrode achieved a current density of 100 mA cm^−2^ at 368 mV [126]. The protection role of the carbon layer at HCD was also confirmed by Kong et al. [119]. They fabricated a 3D self-branched hierarchical nanostructure composed of ultra-small CoP nanoparticles embedded into N,P-co-doped carbon nanotubes knitted hollow nanowall arrays (CoP@NPCNTs HNWAs) on carbon textiles (CTs) via a carbonization–phosphatization process. It exhibited superior electrocatalytic activity and stability in alkaline solutions [119]. It was capable of running for 200 h under the corresponding potential at 100 mA cm^−2^. The assembled CoP@NPCNTs/CTs||CoP@NPCNTs/CTs electrolyzer achieved a high current density of 200 mA cm^−2^ at a low cell voltage of 1.78 V for OWS [119].

Carbon-encapsulated alloys/oxides/sulfides nanosheets grown on NF also show great potential for alkaline OWS at HCD [17,18]. For instance, trimetallic (Fe, Co, Ni) spinel/carbon/nickel foam (FeCoNiO_x_/C/NF) electrodes with 3D network structures have an extremely low Tafel slope of 21 mV dec^−1^ at a current density of 10 mA cm^−2^ in 1.0 M KOH due to superhydrophilic/superaerophobic properties [127]. The optimal FeCoNiO_x_/C/NF reaches a high current density of 500 mA cm^−2^ at a low overpotential of 325 mV with long-term stability of 250 h.

**Figure 9:**
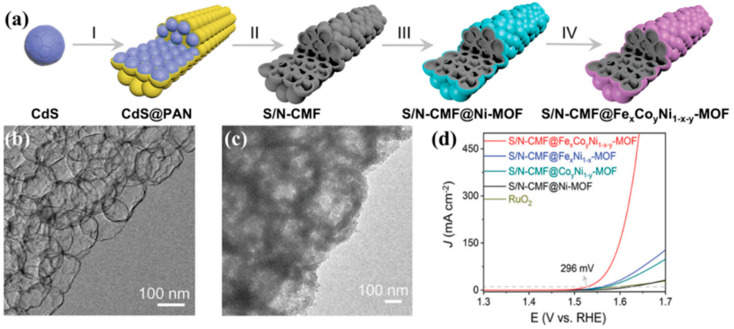
(**a**) Illustration of the synthetic process for creating S/N-CMF@Fe_x_Co_y_Ni_1−x−y_-MOF. TEM images of (**b**) S/N-CMF and (**c**) S/N-CMF@Fe_x_Co_y_Ni_1−x−y_-MOF. (**d**) LSV polarization curves in a 1.0 M KOH solution [68].

In addition to the aforementioned carbon-supported and carbon-encapsulated structures, a reverse carbon-core@shell structure has been developed. A novel OER electrocatalyst of MOFs with ternary metal centers coating S/N-doped carbon mesoporous fibers (S/N-CMF@Fe_x_Co_y_Ni_1−x−y_-MOF) (Figure 9a) has been reported recently [68]. In the last step of the cation exchange reaction, some Ni^2+^ in Ni-MOF was replaced by Fe^2+^/Co^2+^, and the S/N-CMF@Ni-MOF catalyst ultimately transformed into S/N-CMF@Fe_x_Co_y_Ni_1−x−y_-MOF, which retained the original hollow fiber structure. The process was accompanied by the shift of the Ni *d*-band center of Fe/Co-doped γ-NiOOH on the surface of Ni-MOF towards the Fermi level. That is, the doped Fe and Co atoms cooperatively modulated the electronic structure of Ni sites, thereby increasing the number of antibonding states and the energy of valence states and inducing improved OER performances. The enhanced intrinsic activity was conferred by the ternary metal component and the hollow S/N-CMF matrix (Figure 9b,c).

S/N-CMF@Fe_x_Co_y_Ni_1−x−y_-MOF, pasted on CP as the working electrode, exhibits η_10_ and η_200_ of 296 and ~377 mV (Figure 9d), respectively, with a Tafel slope of 53.5 mV dec^−1^.

## 4. Conclusions and Outlook

OER plays a vital role in the performance of a series of green energy storage and conversion devices. The fabrication of cost-effective OER electrocatalysts is highly desirable. This review summarizes the recent progress in carbon-based composites for alkaline OER electrocatalysts from the perspective of design, fabrication, and application.

Some metal-free COF- and POP-based materials, such as TDA-Trz-POP with N- and S-rich surfaces [27], present comparable alkaline OER activity to IrO_2_/RuO_2_. The g-C_3_N_4_ itself is active in OER. As the substrate or shell, it cooperates with the supported or encapsulated materials to boost the OER. Besides g-C_3_N_4_, the widely employed carbon-based materials include biomass-derived porous carbon, rGO, CC, and CNT. Carbon-based materials-supported/encapsulated active materials, e.g., transition metal oxides, hydroxides/LDHs, phosphides, sulfides, selenides, carbides, and alloys, are highly efficient for OER. Structural features, including crystal phases and composition, are closely related to the preparation methods employed, and most of the present carbon-based materials are obtained via high-temperature calcination. A green, affordable, simple, low-cost, and easy large-scale preparation method is urgently needed. Moreover, the thickness of the carbon layer has been confirmed to affect OER activity at both LCD and HCD strongly. Therefore, in addition to assessing the currently used mechanochemically assisted synthesis methods [124] or selecting appropriate types of metal–organic salts [125], future work could focus on developing other facile methods for controlling the thickness of the carbon layer. Convenient methods should also be developed to improve the stability of carbon-based electrocatalysts and avoid degradation [128].

As is well known, the four-electron oxidation reaction involved in OER requires continuous intermediate valence state change steps, making the kinetics of the catalytic cycle very complex and causing slow overall catalytic kinetics [19,129,130,131]. Gaining a deep understanding of the working mechanism of OER electrocatalysts, including the role of carbon-based materials in alkaline OER, is challenging. Future research on in situ experimental techniques and accurate calculation methods to deal with the complex reaction environment is encouraged. An accurate calculation method would include active learning optimization strategies that integrate uncertainty. Various uncertainty factors strongly influence the efficiency of the data-driven method in developing new materials. Therefore, it is important to effectively evaluate uncertainty and use it to develop data-driven strategies to achieve the rational design of new OER electrocatalysts. In addition, developing universal machine learning methods can further save design costs.

To summarize, future work should focus on methods of designing inexpensive electrocatalysts with high activity and stability under complex and harsh reaction conditions.

## Data Availability

Data are contained within the article.
